# Comparative outcomes of balloon-expandable versus self-expanding transcatheter aortic valves: insights from a large-scale German registry

**DOI:** 10.1186/s12872-026-06462-9

**Published:** 2026-08-18

**Authors:** Klaus Kaier, Derek Hazard, Martin Czerny, Jonathan Rilinger, Ingo Hilgendorf, Dennis Wolf, Dirk Westermann, Constantin von zur Mühlen, Christian Valina

**Affiliations:** 1 Department of Cardiology and Angiology, Faculty of Medicine, University Heart Center Freiburg-Bad Krozingen, Medical Center-University of Freiburg, University of Freiburg, Freiburg, Germany; 2 Institute for Medical Biometry and Statistics, Faculty of Medicine and Medical Center, University of Freiburg, Stefan-Meier-Str. 26, Freiburg, Germany; 3 Department of Cardiovascular Surgery, Faculty of Medicine, University Heart Center Freiburg-Bad Krozingen, Medical Center-University of Freiburg, Freiburg, Germany; 4 Freiburg, Germany

**Keywords:** Transcatheter aortic valve replacement, Balloon-expandable valve, Self-expanding valve, Aortic stenosis, Real-world data, Double/debiased machine learning

## Abstract

**Background:**

While transcatheter aortic valve replacement (TAVR) has become an established alternative to surgical aortic valve replacement (SAVR), the comparative outcomes of balloon-expandable (BE) and self-expanding (SE) valve technologies remain an area of ongoing investigation.

**Methods:**

Using the German DESTATIS database, we analyzed 48,565 transfemoral TAVR procedures performed between 2021 and 2022. Outcomes were compared between BE (*n* = 23,412) and SE (*n* = 25,153) valves using a double/debiased machine learning estimator to account for potential confounding. Key endpoints included in-hospital mortality, major bleeding, stroke, acute kidney injury, mechanical ventilation > 48 h, postoperative delirium, permanent pacemaker implantation (PPI), length of hospital stay, and reimbursement.

**Results:**

Descriptively, SE valves were associated with significantly lower in-hospital mortality (1.6% vs. 2.0%, *p* < 0.001) and major bleeding (1.4% vs. 2.1%, *p* < 0.001) but a higher risk of stroke (2.4% vs. 1.9%, *p* < 0.001). A trend towards higher risk of PPI was observed (*p* = 0.078). After adjustment, patients with SE valves are at lower risk for in-hospital mortality (RR 0.85, *p* = 0.049) and major bleeding (RR 0.78, *p* = 0.006), but had a higher risk of stroke (RR 1.35, *p* < 0.001) and permanent pacemaker implantation (RR 1.09, *p* = 0.028). Subgroup analysis indicated that older and high-risk patients, particularly those aged ≥ 85 years, showed a stronger association with lower in-hospital mortality for SE valves, whereas younger patients exhibited a trend favouring BE valves. BE valves were also associated with a modestly higher reimbursement (€209 per case, *p* = 0.004).

**Conclusion:**

In this large, real-world cohort, self-expanding valves were associated with lower in-hospital mortality and major bleeding but a higher risk of stroke and permanent pacemaker implantation compared with balloon-expandable valves. Because prosthesis choice in routine practice is highly individualized and driven by anatomical and procedural factors not captured in administrative data, these associations are hypothesis-generating and cannot support device-selection recommendations.

**Supplementary Information:**

The online version contains supplementary material available at 10.1186/s12872-026-06462-9.

## Introduction

Transcatheter aortic valve replacement (TAVR) has been described to be advantageous compared to surgical aortic valve replacement (SAVR) in a variety of patients suffering from aortic valve stenosis. Major landmark studies are mainly industry-sponsored and therefore focus either on outcomes of balloon-(BE) or self-expandable (SE) technologies [1]. The recently published DEDICATE study allowed choice of valve type depending upon the decision of the local team [2], and results were favorable among patients with low or intermediate surgical risk undergoing TAVR after 1 year with respect to death from any cause or stroke. However, with 1414 patients, this study was too small to define differences in outcome depending upon the valve type used. Large national patient databases could therefore help to elucidate this open question, which is important in the widening indication for TAVI, reaching from low to high risk patients [3]. Given the increasing use of TAVR in younger and lower-risk populations, understanding potential differences between valve types is essential for optimizing patient outcomes. Additionally, real-world data from national registries can complement randomized trial findings by providing insights into everyday clinical practice. Building on our previous nationwide German comparison of the 2019/2020 period [3], the present analysis examines whether balloon- and self-expanding platforms differ in in-hospital outcomes in the more recent, higher-volume 2021/2022 cohort, using a doubly robust estimator. Randomized comparisons such as CHOICE [4], SCOPE I [5], and SOLVE-TAVI [6] were conducted with earlier-generation devices and were underpowered to detect differences in low-frequency in-hospital endpoints, leaving this question open in contemporary unselected practice.

In this context, our study aims to contribute valuable evidence regarding the comparative effectiveness of BE and SE valves in a contemporary German patient cohort, applying a doubly robust machine learning approach to account for measured differences in patient risk profiles between the two valve groups.

## Methods

Since its inception in 2005, the DESTATIS research data center, operating under the auspices of the German Federal Statistical Office, has been facilitating access to an extensive repository of data on hospitalization trends from all hospitals across Germany. These insights are predominantly sourced from inpatient billing databases integrated within the German Diagnosis-Related Groups (DRG) framework. Notably, the DRG system employs a nuanced classification methodology, wherein costs are aggregated into discrete groups based on two key factors: diagnosed medical conditions, encoded in accordance with the globally recognized ICD-10 (International Classification of Diseases, 10th Revision) protocol, and performed clinical interventions, cataloged using the German operation and procedure classification system (OPS). The unit of analysis is the hospital admission (procedure). DESTATIS provides anonymized case-level records without a cross-admission patient identifier; individual patients cannot be linked across admissions, so a small number of repeat admissions may be present. Admissions with concurrent codes for more than one valve platform, a second valve implantation, or ambiguous device coding were excluded. The complete inclusion/exclusion algorithm with ICD-10 and OPS codes and a patient-selection flow diagram are provided in Supplemental Table S2.

We extracted information on 48,565 TAVR cases performed in 2021 and 2022, representing the most recent years for which complete data were available at the time of analysis. The analysis focused on nine pre-specified endpoints, defined as in our previous work [3]: in-hospital mortality, claims-defined major bleeding, stroke, acute kidney injury, postoperative delirium, mechanical ventilation exceeding 48 hours, permanent pacemaker implantation (PPI), hospital length of stay and reimbursement (CPI adjusted for 2022). The subgroup analyses of in-hospital mortality are exploratory. Because administrative data record only whether an event occurred during the admission, non-fatal endpoints are ‘ever-during-hospitalization’ outcomes with unknown sequence relative to death. In detail, acute kidney injury was identified using ICD-10 codes, specifically the secondary diagnosis code N17. Major bleeding events were defined by the necessity for transfusion of more than five units of red blood cells and were identified using OPS codes (8-800.c1 to 8-800.cr). This transfusion-based definition is restrictive and not equivalent to VARC-3 bleeding categories; it may not capture fatal or intracranial bleeding without large-volume transfusion or access-site bleeding managed by intervention. Administrative data indicate whether, but not when, an event occurred during the admission; non-fatal endpoints are therefore “ever-during-hospitalization” outcomes. The primary variables included in the DESTATIS dataset were in-hospital mortality, duration of mechanical ventilation, hospital length of stay, and reimbursement. To calculate an estimated logistic EuroSCORE (EuroSCORE I, logistic version), all components could be populated from the dataset except ‘critical preoperative state’ and ‘left ventricular function’, which are not coded in DESTATIS. Critical preoperative state was set to absent for all patients; for left ventricular function a fixed intermediate assumption was applied, with moderate dysfunction (LVEF 30–50%) assigned to all patients and poor dysfunction (LVEF < 30%) to none [7]. The variant used additionally sets the factor ‘other than isolated CABG’ to absent for all cases. Because these are constants applied identically to both groups, they alter the absolute level of the score but not the comparison between platforms. Since the measure is therefore not the fully validated logistic EuroSCORE, it is labelled ‘estimated logistic EuroSCORE’ throughout; the full derivation, including every component, its regression coefficient and its ICD-10-GM source, is given in Supplemental Table S2.

Differences in baseline characteristics and outcomes between groups were assessed using the Student’s t-test for continuous variables and the chi-square test for categorical variables. To account for potential selection bias in treatment assignment, we estimated the average treatment effect using a double/debiased machine learning (DML) estimator [8]. Given the large number of potential confounders, DML employs machine learning algorithms to flexibly model the relationships between covariates and both the outcome and treatment variables. For variable selection, we applied the adaptive lasso (least absolute shrinkage and selection operator), a machine learning technique known for its oracle property, which enhances the identification of relevant variables [9]. Specifically, we used the telasso program in Stata, which integrates adaptive lasso with propensity score-based augmented inverse probability weighting (AIPW). The procedure fits separate outcome models for the treated and untreated potential-outcome means together with a logistic treatment (propensity) model and combines them by augmented inverse-probability weighting. Because this method relies on either—but not necessarily both—models being correctly specified, it is referred to as a “doubly robust” estimation technique [10–12]. All of the following 21 candidate confounders were supplied to both the outcome and treatment (propensity) models [3, 13]: (1) age, (2) female sex, (3) estimated logistic EuroSCORE, (4) Hospital Frailty Risk Score, (5) NYHA class III/IV, (6) coronary heart disease, (7) arterial hypertension, (8) previous myocardial infarction within 4 months, (9) previous myocardial infarction within 1 year, (10) previous myocardial infarction after more than 1 year, (11) previous CABG, (12) previous cardiac surgery (EuroSCORE component), (13) peripheral atherosclerotic disease, (14) carotid disease, (15) COPD, (16) pulmonary hypertension, (17) renal disease with GFR < 15, (18) renal disease with GFR 15–30, (19) atrial fibrillation, (20) diabetes, and (21) emergency admission. All comorbidities were identified from ICD-10 secondary-diagnosis codes as defined in our previous work [3]; for example, pulmonary hypertension (I27.0, I27.2-) and COPD (J44.-). Adaptive lasso was allowed to penalize (i.e., potentially exclude) any of these covariates from each nuisance model; the variables retained in every model are reported in Supplemental Table S1. Using this doubly robust estimator, we first estimated potential outcome means and subsequently derived adjusted risk ratios by the delta method. Because treatment was not randomly allocated, these quantities are interpreted as covariate-adjusted associations, not causal effects. Cluster robust standard errors at the hospital level were specified to accommodate the correlation of error terms among patients treated at the same hospital. For the treatment models, an overlap plot was constructed and checked whether the overlap assumption is violated. The overlap assumption is satisfied when there is a chance of seeing observations in both groups at each combination of covariate. The overlap (positivity) assumption was assessed from the distribution of estimated treatment probabilities (see Supplemental Table S1). Although the number of predefined confounders is modest relative to the sample size, we employed this machine learning approach for variable selection as it is an integral part of the DML framework, which uses regularization to prevent overfitting in the nuisance models and thereby ensures robust inference.

Missing values could not be formally imputed, as the administrative data does not use specific codes to indicate missing data. Therefore, in cases where a code for a specific clinical characteristic was absent from a patient’s record, it was presumed that the characteristic was not present. The frequency of such implicit missingness cannot be precisely quantified, which is a known limitation of this type of registry data. We did not adjust for multiple testing. As a result, the p-values should be regarded as descriptive rather than confirmatory, and any conclusions drawn from the 95% confidence intervals might not be consistently replicable. The analyses were conducted using Stata 18, a software developed by StataCorp, located in College Station, Texas, USA. Patients and the public were not involved in the design, conduct, or reporting of this research, given its reliance on a pre-existing administrative dataset.

## Results

Overall, 48,565 transfemoral TAVR procedures with 23,412 balloon-expandable and 25,153 self-expanding valves. Patients in the SE group had a higher estimated logistic EuroSCORE of 13.2 vs. 12.1% in BE (*p* < 0.001), were of higher age (81.77 vs. 80.23, *p* < 0.001), were more often female (54.8 vs. 39.2%, *p* < 0.001), and more often had the comorbidities heart failure (48.9 vs. 47.5%, *p* = 0.002), arterial hypertension (64.5 vs. 62.6%, *p* < 0.001) and pulmonary hypertension (17.1 vs. 16.4%, *p* = 0.035). On the other side, patients in the BE group had a higher frailty score (0.27 vs. 0.21, *p* < 0.001) and they had more often comorbidities such as previous myocardial infarction (6.7 vs. 5.0%, *p* < 0.001), previous cardiac surgery (13.9 vs. 12.2%, *p* < 0.001), carotid disease (6.2 vs. 5.6%, *p* = 0.007) and emergency admissions (10.3 vs. 9.7%, *p* = 0.043). Because of the large sample, p-values are of limited value for judging balance; absolute standardized mean differences exceeded 0.10 only for female sex (0.32), age (0.25), calendar year (0.14), and the estimated logistic EuroSCORE (0.12), while all other covariates were well balanced (Table 1).

**Table 1:** Baseline characteristics

	Overall	BE-valves	SE-valves	*P*-Value	SMD
*N*	48,565 (100.0%)	23,412 (48.2%)	25,153 (51.8%)		
Age in years, mean (SD)	81.03 (6.26)	80.23 (6.65)	81.77 (5.79)	< 0.001	0.25
Female, N (%)	22,961 (47.3%)	9,169 (39.2%)	13,792 (54.8%)	< 0.001	0.32
Estimated logistic EuroSCORE, mean (SD)	12.69 (9.44)	12.10 (9.30)	13.23 (9.54)	< 0.001	0.12
Frailty Score, mean (SD)	0.24 (0.96)	0.27 (0.98)	0.21 (0.93)	< 0.001	0.06
NYHA III or IV, N (%)	23,417 (48.2%)	11,116 (47.5%)	12,301 (48.9%)	0.002	0.03
CAD, N (%)	24,740 (50.9%)	12,183 (52.0%)	12,557 (49.9%)	< 0.001	0.04
Hypertension, N (%)	30,886 (63.6%)	14,652 (62.6%)	16,234 (64.5%)	< 0.001	0.04
Previous MI within 4 months, N (%)	725 (1.5%)	417 (1.8%)	308 (1.2%)	< 0.001	0.05
Previous MI 4 months to 1 year, N (%)	295 (0.6%)	161 (0.7%)	134 (0.5%)	0.028	0.03
Previous MI after 1 year, N (%)	1,819 (3.7%)	989 (4.2%)	830 (3.3%)	< 0.001	0.05
Previous CABG, N (%)	3,451 (7.1%)	1,699 (7.3%)	1,752 (7.0%)	0.211	0.01
Previous cardiac surgery, N (%)	6,323 (13.0%)	3,244 (13.9%)	3,079 (12.2%)	< 0.001	0.05
Peripheral vascular disease, N (%)	4,313 (8.9%)	2,085 (8.9%)	2,228 (8.9%)	0.853	0.00
Carotid disease, N (%)	2,844 (5.9%)	1,441 (6.2%)	1,403 (5.6%)	0.007	0.03
COPD, N (%)	4,196 (8.6%)	2,007 (8.6%)	2,189 (8.7%)	0.610	0.00
Pulmonary hypertension, N (%)	8,133 (16.7%)	3,834 (16.4%)	4,299 (17.1%)	0.035	0.03
Renal disease, GFR < 15, N (%)	1,021 (2.1%)	552 (2.4%)	469 (1.9%)	< 0.001	0.03
Renal disease, GFR 15–30, N (%)	1,770 (3.6%)	827 (3.5%)	943 (3.7%)	0.203	0.01
Atrial fibrillation, N (%)	21,312 (43.9%)	10,266 (43.8%)	11,046 (43.9%)	0.884	0.00
Diabetes, N (%)	14,832 (30.5%)	7,243 (30.9%)	7,589 (30.2%)	0.067	0.02
Emergency, N (%)	4,850 (10.0%)	2,405 (10.3%)	2,445 (9.7%)	0.043	0.02
Year					
2021	23,292 (48.0%)	10,365 (44.3%)	12,927 (51.4%)	< 0.001	0.14
2022	25,273 (52.0%)	13,047 (55.7%)	12,226 (48.6%)		

Abbreviations: *BE* balloon-expandable, *CABG* coronary artery bypass grafting, *CAD* coronary artery disease, *COPD* chronic obstructive pulmonary disease, *EuroSCORE* European System for Cardiac Operative Risk Evaluation, *GFR* glomerular filtration rate, *ICD-10-GM* International Statistical Classification of Diseases and Related Health Problems, 10th Revision, German Modification, *MI* myocardial infarction, *N* number of observations, *NYHA* New York Heart Association, *SD* standard deviation, *SE* self-expanding, *SMD* standardized mean difference

Patients in the BE group had a significantly higher rate of in-hospital mortality (2.0 vs. 1.6%, *p* < 0.001) major bleeding (2.1 vs. 1.4%, *p* < 0.001), acute kidney injury (9.0 vs. 8.3%, *p* = 0.008), mechanical ventilation > 48 h (1.6 vs. 1.1%, *p* < 0.001), a longer length of hospital stay (10.4 vs. 9.9 days, *p* < 0.001) and were associated with higher reimbursements (€26,663 vs. €26,306, *p* < 0.001). In contrast, the SE group showed a higher rate of stroke (2.4 vs. 1.9%, *p* < 0.001). No significant difference was observed for postoperative delirium (*p* = 0.181), and there was a non-significant trend toward higher permanent pacemaker implantation in the SE group (*p* = 0.078).

**Fig. 1:**
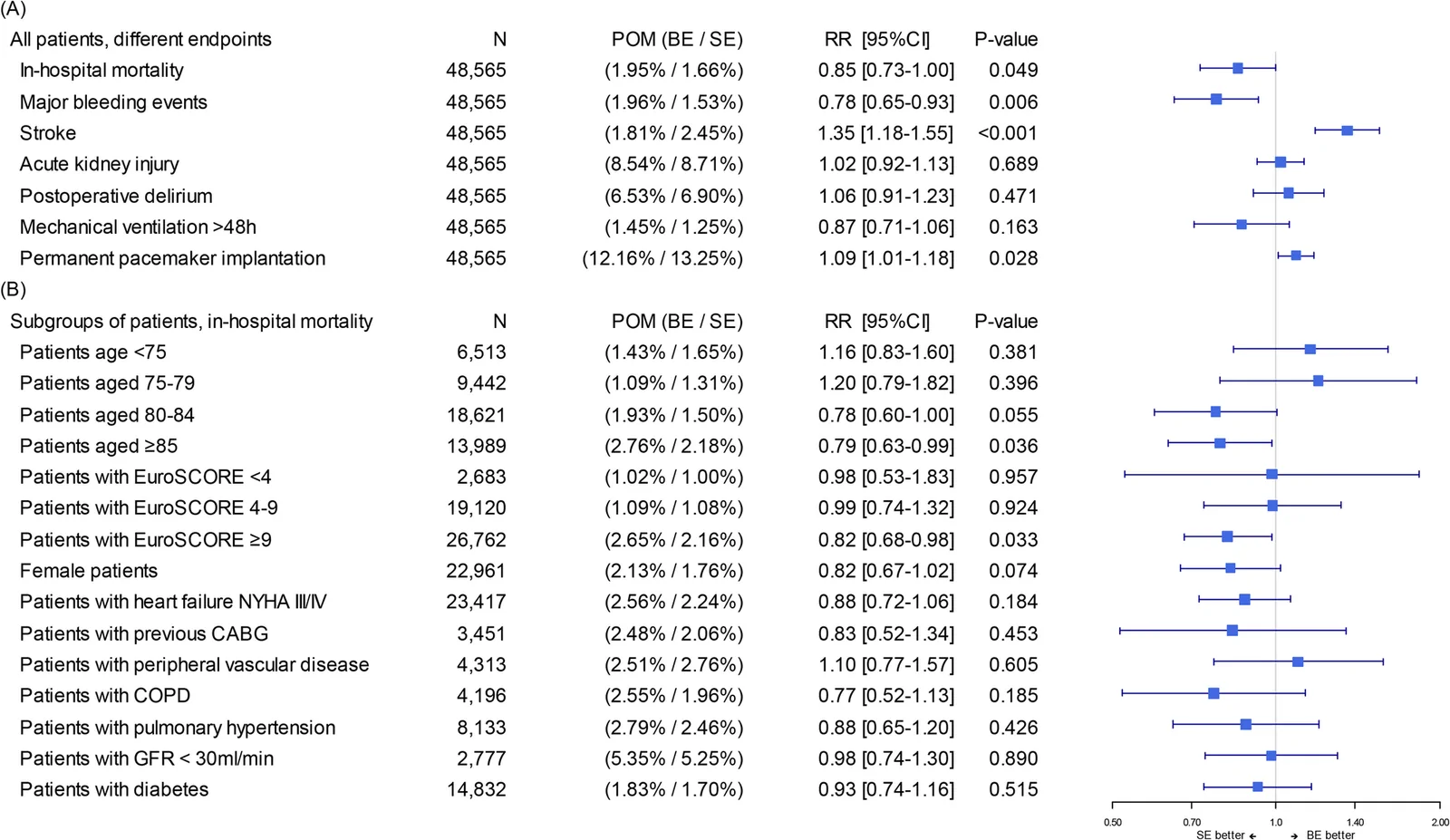
Results of the causal analyses regarding different in-hospital endpoints (**A**) or within subgroups of patients for the endpoint in-hospital mortality (**B**). Predicted outcome means (POM) were calculated based on the doubly robust approach and may be interpreted as the expected outcome if all individuals in the (sub) population had received the respective treatment. Based on these values, the average treatment effect was calculated as causal risk ratio (RR)

The descriptive rates in Table 2 are unadjusted. After adjustment for the 21 covariates using the doubly robust estimator (Fig. 1A), the direction of the main associations was preserved: Patients with SE valves are at lower risk for in-hospital mortality (RR 0.85, *p* = 0.049) and major bleeding (RR 0.78, *p* = 0.006), but had a higher risk of stroke (RR 1.35, *p* < 0.001) and permanent pacemaker implantation (RR 1.09, *p* = 0.028). Notably, even though the SE group had higher rates of costly complications like stroke and PPI, reimbursement was still slightly higher for the BE group (€209 difference per case, *p* = 0.004), while no significant difference was identified for the length of hospital stay (SE-valves could be discharged 0.38 days earlier; *p* = 0.103). The cluster-robust variance estimation was based on 84 hospital institution codes (IKs). Because an IK may change following a change in a hospital’s legal form, this number may slightly exceed the number of distinct physical centres and should not be interpreted as an exact hospital count.

**Table 2:** In-hospital outcomes

	Overall	BE-valves	SE-valves	*P*-value
*N*	48,565 (100.0%)	23,412 (48.2%)	25,153 (51.8%)	
In-hospital mortality, N (%)	864 (1.8%)	466 (2.0%)	398 (1.6%)	< 0.001
Major bleeding events, N (%)	841 (1.7%)	486 (2.1%)	355 (1.4%)	< 0.001
Stroke, N (%)	1,035 (2.1%)	437 (1.9%)	598 (2.4%)	< 0.001
Acute kidney injury, N (%)	4,188 (8.6%)	2,101 (9.0%)	2,087 (8.3%)	0.008
Postoperative delirium, N (%)	3,225 (6.6%)	1,518 (6.5%)	1,707 (6.8%)	0.181
Mechanical ventilation > 48 h, N (%)	655 (1.3%)	380 (1.6%)	275 (1.1%)	< 0.001
Permanent pacemaker implantation, N (%)	6,158 (12.7%)	2,904 (12.4%)	3,254 (12.9%)	0.078
Length of hospital stay, in days, mean (SD)	10.16 (7.19)	10.40 (7.75)	9.94 (6.63)	< 0.001
Reimbursement, in €, mean (SD)	26478.61 (4371.97)	26663.43 (4893.53)	26306.58 (3815.16)	< 0.001

Subgroup analysis indicated that older and high-risk patients, particularly those aged 80–84 years (RR 0.78, *p* = 0.055), aged ≥ 85 years (RR 0.79, *p* = 0.036) and patients with a EurioSCORE ≥ 9 (RR 0.82, *p* = 0.033), showed a stronger association with lower in-hospital mortality for SE valves. At the same time, younger patients exhibited a trend favouring BE valves (see Fig. 1B). BE valves were also associated with a higher reimbursement (€209 per case, *p* = 0.004) according to the applied approach.

## Discussion

### Principal findings

Overall, our findings indicate that patients receiving SE-valves experienced a significantly lower risk of in-hospital mortality and major bleeding, but a higher risk of stroke and permanent pacemaker implantation (PPI) compared to those receiving BE-valves. Our analysis of baseline characteristics revealed that patients receiving SE valves were generally older and had a higher comorbidity burden, though surprisingly, the prevalence of previous cardiac surgery, a factor sometimes favoring BE valves for easier coronary access, was only slightly higher in the BE group. Additionally, our subgroup analysis suggests that older patients, particularly those 85 years and older, appear to benefit more from SE valves in terms of in-hospital mortality, whereas younger patients may have better outcomes with BE valves. Furthermore, reimbursement was higher for BE valves despite no significant difference in hospital length of stay.

### Comparison with prior evidence

Our observation of lower in-hospital mortality with SE valves contrasts with several studies that report similar mortality rates between the two valve types, although many of these analyses focus on 30-day or 1-year mortality rather than the in-hospital endpoint used here [14–16]. When focusing on the event rate during short term follow-up in randomized studies the 30-day mortality rate was 4.1% in the BE valve group and 5.1% in the SE valve group (RR 0.81, 95%CI 0.25–2.57; *p* = 0.77) in the CHOICE-trial [4] and 1% vs. 2% (*p* = 0.09) in the SCOPE I-trial [5]. Thiele et al. showed a 30-day mortality rate of 3.2% in the SE valve group vs. 2.3% in the BE valve group (Rate difference − 0.94, 90%CI -4.79-2.91) in the SOLVE-TAVI-trial [6]. These randomized trials report 30-day and 1-year outcomes, whereas the present study reports in-hospital events; the different follow-up windows preclude direct numerical comparison and the trial figures are provided for context only.

The finding of a lower risk of major bleeding with SE valves is not consistently supported by the literature. Our administrative data does not allow for differentiation of bleeding sources (e.g., access site vs. internal) or analysis of concomitant medications like anticoagulants, which could influence these rates. In fact, many studies report no significant difference in bleeding events between SE and BE valves [17, 18]. The procedure related major bleeding rate in the SOLVE-TAVI-trial was 3,2% in the BE valve group and 2,3% SE valve group (*p* = 0,56) [6]. In randomized studies the 30-day bleeding rate for major bleeding was 19.0% in the BE valve group and 14.5% in the SE valve group (RR 1.31, 95%CI 0.74–2.32; *p* = 0.36) in the CHOICE-trial [4] and 9.0% vs. 11.0% (*p* = 0,21) in the SCOPE I-trial [5].

The higher risk of stroke associated with SE-valves is supported by some studies [16, 19], while others report no significant difference [14, 17, 20]. Focusing again on the event rate during short term follow-up in randomized studies the 30-day stroke rate was 5.8% in the BE valve group and 2.6% in the SE valve group (RR 2.26, 95%CI 0.60–8.52; *p* = 0.33) in the CHOICE-trial [4] and 3.0% vs. 2.0% (*p* = 0.33) in the SCOPE I-trial [5]. But the stroke rate in the SOLVE-TAVI-trial was significantly lower in the SE valve group (0.5% vs. 4.7%, Rate difference 4.2, 90%CI 0.11–8.28) [6]. The underlying reasons remain debated, and our data lacks procedural details, such as rates of pre- or post-dilatation, that could contribute to this risk. Some data suggests that a longer implant time and increased manipulation of calcified tissue, particularly with SE valves, can lead to higher embolization risk and subsequent stroke [19].

The recently in August 2025 published new guidelines for the management of valvular heart disease just mention adverse event rates e.g. new pacemaker implantations are more frequent after TAVI, particularly when using SE valves. But they do not recommend any specific type of TAVI for each patient with severe aortic valve stenosis. The new guidelines leave this decision to the Heart Team, which decides for BE valve or SE valve according clinical characteristics, vessel access, valve anatomy and repeat procedure options and risks (lifetime management) [21]. As the guidelines deliberately refrain from recommending a specific prosthesis type [21], our findings are consistent with and complement this framework, but—being derived from administrative data without anatomical or procedural detail—cannot themselves establish device-specific treatment recommendations.

We also identified higher reimbursement for BE valves. This reimbursement observation is not directly supported by the literature, although some data suggests that the length of stay is longer in the BE group [22]. This finding regarding reimbursement could have a major implication for health economic decision-making. The adjusted reimbursement difference of €209 per case (*p* = 0.004) is modest—below 1% of mean DRG reimbursement—and is itself largely driven by complication coding, and therefore subject to the same residual confounding as the clinical endpoints. German DRG reimbursement reflects case-mix classification and coding rules rather than true hospital cost, device acquisition price, or cost-effectiveness, and does not capture rehabilitation, readmission, or post-discharge care. This finding should therefore not be interpreted as demonstrating economic superiority of either platform. In addition, the reimbursement observations are specific to the German DRG system and its coding and pricing rules, and should not be generalised to health systems with different financing structures.

Of interest is the subgroup analysis of in-hospital mortality (Fig. 1B). While “younger” patients overall show a tendency towards improved mortality with BE-valves, older patients and especially those aged 85 years and older seem to significantly benefit from SE-valves – an aspect that, to our knowledge, has not been previously described in such a big data cohort. The same is true for patients with a high risk according to the EuroSCORE. The exact reasons for this finding remain speculative – from potentially reduced rapid pacing rates in SE patients as a benefit in older patients, and reduced pacemaker rates in BE patients of younger age. However, due to the large number of patients included in our study, the reduced mortality rate in elderly patients with SE-TAVI is considered to be a significant finding with potential impact on clinical decision making in this vulnerable subset of patients.

### Limitations

Our research design constraints present additional challenges beyond the usual scope of retrospective study designs [7, 23]. Notably, its foundation in administrative data introduces inherent vulnerabilities, primarily the inevitability of coding inaccuracies. Furthermore, the risk adjustment integrated into our model may not capture the full spectrum of pertinent factors, as the reliability of some included parameters is open to question. An additional constraint stems from the administrative dataset’s lack of critical clinical insights, such as echocardiogram findings and anatomical specifics, which hampers a nuanced evaluation of surgical risks and a deeper elucidation of valvular dysfunction mechanisms. As a result, we employ a surrogate, conservative application of the estimated logistic EuroSCORE, effectively yielding a ‘best-case’ scenario approximation. Because roughly half of the cohort was in NYHA class III/IV, the assumption of normal left ventricular function for all patients when reconstructing the EuroSCORE is optimistic and probably underestimates operative risk. NYHA class III/IV was nevertheless retained as a separate covariate in all adjustment models, partially preserving functional-status information. Crucially, several determinants of both prosthesis selection and early outcome are entirely absent from administrative data, including left ventricular ejection fraction, critical preoperative status, annular and root anatomy, degree and distribution of calcification, coronary access considerations, and procedural details such as access route, type of anaesthesia, and pre- or post-dilatation. Because these variables are unmeasured, adjustment—however sophisticated—cannot remove the associated confounding, and substantial residual confounding, including confounding by indication, is likely. The reported estimates should therefore be interpreted as adjusted associations rather than treatment effects. The analysis was restricted to transfemoral procedures, so access route is not a source of confounding within this analysis. In addition, selection of prosthesis type by local experience, institutional preference, and procurement or contractual arrangements cannot be measured in administrative data and may contribute to residual confounding. The study’s scope is also delimited by the exclusion of patients presenting with pre-existing aortic regurgitation, as well as those undergoing TAVR in conjunction with other cardiac procedures. While clinically justifiable, this approach complicates direct comparisons with external administrative datasets. An additional layer of complexity arises from the absence of detailed device-specific information within our dataset, extending only to device type. For example, within the realm of self-expanding (SE) valves, the ACURATE neo [24] and the CoreValve [25] are in use in Germany [26], yet our data does not capture such granular distinctions. The findings therefore apply to heterogeneous platform categories used in Germany during 2021–2022 and must not be read as a direct comparison of specific contemporary devices. Finally, our analysis could not account for the competing risk of death, which may be particularly relevant when comparing non-fatal outcomes. For instance, higher early mortality in one group could preclude the occurrence of a later event like stroke or the need for PPI. A competing risks analysis was not feasible as the administrative dataset lacks the detailed event timing required for such modeling. As an observational analysis without randomized treatment allocation, this study cannot establish causality, comparative cost-effectiveness, or a preferred valve platform for individual patients.

## Conclusion

In this nationwide administrative cohort, self-expanding valves were associated with lower in-hospital mortality and claims-defined major bleeding but higher stroke and pacemaker rates than balloon-expandable valves; the mortality association was borderline (adjusted RR 0.85, 95% CI 0.73–1.00, *p* = 0.049) and most apparent in elderly and high-risk patients. Given the observational design and the absence of key anatomical, functional, and procedural data, these associations are hypothesis-generating, cannot establish causality or a preferred prosthesis for individual patients, and should be interpreted with caution pending confirmation in adjudicated datasets.

## Supplementary Information

Supplementary Material 1.

Supplementary Material 2.

## Data Availability

The data that support the findings of this study are available from the Research Data Centre of the Federal Statistical Office of Germany (Forschungsdatenzentrum der Statistischen Ämter des Bundes und der Länder, DESTATIS), but restrictions apply to the availability of these data, which were used under a data use agreement for the current study and are therefore not publicly available. Access to the data can be obtained by qualified researchers upon application to the Research Data Centre, subject to its data protection regulations and the relevant German legal framework. Further information is available at https://www.forschungsdatenzentrum.de/.
