# Supplementary material for: Comparative outcomes of balloon-expandable versus self-expanding transcatheter aortic valves: insights from a large-scale German registry
Source: BMC Cardiovasc Disord. 2026 Aug 18;26:723. doi: 10.1186/s12872-026-06462-9 (PMC13488022; doi:10.1186/s12872-026-06462-9)

Table S1: Selected variables using the lasso for the outcome in-hospital mortality

	Outcome model		Treatment model
	BE-valves	SE-valves	
Age in years	x		x
Logistic EuroSCORE	x	x	
Frailty Score	x	x	x
Previous MI within 4 months	x		
COPD	x		
Renal disease, GFR <15	x		
Diabetes	x		
Emergency	x		x
Female			x
Constant	x	x	x

Figure S1: Overlap plot

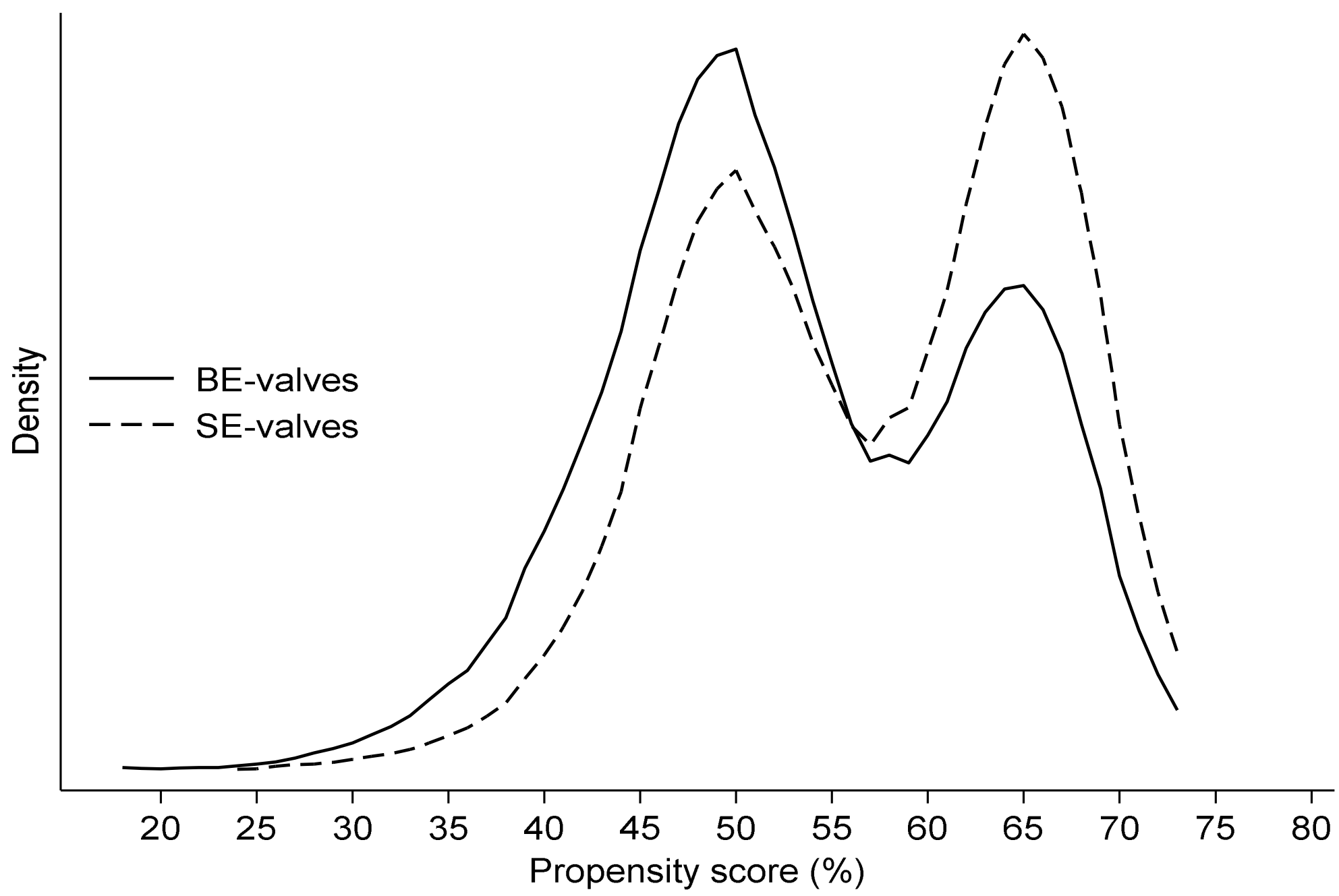

Supplement: Supplementary file 1 [file 12872_2026_6462_MOESM1_ESM.pdf]
