# Supplementary material for: Comparative outcomes of balloon-expandable versus self-expanding transcatheter aortic valves: insights from a large-scale German registry
Source: BMC Cardiovasc Disord. 2026 Aug 18;26:723. doi: 10.1186/s12872-026-06462-9 (PMC13488022; doi:10.1186/s12872-026-06462-9)
Supplement: Supplementary file 2 [file 12872_2026_6462_MOESM2_ESM.pdf]

Table S2: Coding details

Inclusion: Aortic valve stenosis	ICD-10-GM	I350, I352, I060, I062
Inclusion: balloon-expandable (BE)	OPS	535a03, 535b11
Inclusion: self-expanding (SE)	OPS	535a04, 535b12
Exclusion: other concomitant procedures	OPS	535101, 535102, 535103, 535104, 53511*, 53512*, 53531*, 53532*, 53541*, 53514*, 5361*, 5362*, 5363*, 883700, 883701, 8837k*, 8837m*

Baseline characteristics

Age (years)	DESTATIS-Variable	
Female sex	DESTATIS-Variable	
Estimated logistic EuroSCORE (in %)	Calculated based on [4], see below	
Hospital Frailty Risk Score (deutsche Rekalibrierung)	Calculated based on [10]	
NYHA class III/IV	ICD-10-GM	I5013, I5014
Coronary heart disease	ICD-10-GM	I2511, I2512, I2513
Arterial hypertension	ICD-10-GM	I10*
Previous myocardial infarction within 4 months	ICD-10-GM	I2520
Previous myocardial infarction within 1 year	ICD-10-GM	I2521
Previous myocardial infarction more than 1 year ago	ICD-10-GM	I2522
Previous CABG	ICD-10-GM	Z951*
Previous cardiac surgery	ICD-10-GM	Z951, Z952, Z953, Z954
Peripheral vascular disease (Baseline-Variante)	ICD-10-GM	I7020-I7025, I708, I709, I739
Carotid disease	ICD-10-GM	I652
COPD	ICD-10-GM	J44*
Pulmonary hypertension	ICD-10-GM	I27*
Renal disease, GFR < 15 ml/min	ICD-10-GM	N185
Renal disease, GFR 15-30 ml/min	ICD-10-GM	N184
Atrial fibrillation	ICD-10-GM	I480, I481, I482, I489
Diabetes mellitus	ICD-10-GM	E10*, E11*, E12*, E13*, E14*
Emergency admission	DESTATIS-Variable	

Endpoints

In-hospital mortality	DESTATIS-Variable	
Claims-defined major bleeding (mehr als 5 Erythrozytenkonzentrate)	OPS	8800c1, 8800c2, 8800c3 to 8800cr
Stroke	ICD-10-GM	I63*, I64
Acute kidney injury	ICD-10-GM	N17*
Postoperative delirium	ICD-10-GM	F05*
Mechanical ventilation > 48 hours	DESTATIS-Variable	
Permanent pacemaker implantation	OPS	53770, 53771, 53772, 53773*, 53774*, 53775*, 53776, 53777*, 5377j
Length of hospital stay (days)	DESTATIS-Variable	
Reimbursement (EUR, cpi-adjusted for 2022)	DESTATIS-Variable	

**Estimated logistic EuroSCORE**	**Beta-coefficient**	**Code or source**
Age	0.0666354	DESTATIS-Variable
Sex (female)	0.3304052	DESTATIS-Variable
Chronic pulmonary disease	0.4931341	J44*, J43*
Extracardiac arteriopathy	0.6558917	I702*, I708, I709, I739, I652
Neurological dysfunction	0.8416260	I69*, G81*, R48*
Previous cardiac surgery	1.0026250	Z951, Z952, Z953, Z954
Serum creatinine > 200 µmol/l	0.6521653	N184, N185
Active endocarditis	1.1012650	I33*
Unstable angina	0.5677075	I200
Recent myocardial infarct	0.5460218	I2520
Pulmonary hypertension	0.7676924	I27*
Emergency	0.7127953	DESTATIS-Variable
